# CD11b expression on monocytes and data of inflammatory parameters after Transcatheter Aortic Valve Implantation in dependence of early mortality

**DOI:** 10.1016/j.dib.2020.105798

**Published:** 2020-06-02

**Authors:** C. Pfluecke, S. Wydra, K. Berndt, D. Tarnowski, M. Cybularz, P. Barthel, A. Linke, K. Ibrahim, DM. Poitz

**Affiliations:** 1 Technische Universität Dresden, Heart Center Dresden, University Hospital, Germany; 2 Department of Cardiology, Universitätsklinikum Leipzig, Leipzig, Germany; 3 Department of Internal Medicine II, University Hospital Regensburg, Regensburg, Germany; 4 Department of Cardiology, Technische Universität Dresden, Klinikum Chemnitz, Chemnitz, Germany.; 5 Institute for Clinical Chemistry and Laboratory Medicine, Faculty of Medicine, Technische Universität Dresden, Germany

**Keywords:** Transcatheter Aortic Valve Implantation, inflammation, monocytes, CD11b expression, Monocyte-subsets, Monocyte-platelet-aggregates

## Abstract

An inflammatory systemic reaction is common after Transcatheter Aortic Valve Implantation (TAVI). We recently reported about an involvement of Mon2-monocytes, the CD11b expression on monocytes and parameters of systemic inflammation before TAVI correlating with early mortality after TAVI. Here, we provide data of monocyte subpopulations, CD11b expression and parameters of a systemic inflammation in dependence of three-month mortality after TAVI. With this, we provide further insights into inflammatory mechanism after TAVI. The data were collected by flow-cytometric quantification analyses of peripheral blood in 120 consecutive patients who underwent TAVI (on day 1 and 7 after TAVI). Monocyte-subsets were identified by their CD14 and CD16 expression and monocyte-platelet-aggregates (MPA) by CD14/CD41 co-expression. The extent of monocyte activation was determined by quantification of CD11b-expression (activate epitope). Additionally, pro-inflammatory cytokines such as interleukin (IL)-6, IL-8, C-reactive protein, procalcitonin were measured using the cytometric bead array method or standard laboratory tests. Additionally, we report procedural outcomes in dependence of three-month mortality. Furthermore, correlations of CD11b-expression on monocytes with parameters of platelet activation or further inflammatory parameters are presented. For further interpretation of the presented data, please see the research article “Mon2-Monocytes and Increased CD-11b Expression Before Transcatheter Aortic Valve Implantation are Associated with Earlier Death” by Pfluecke et al.[1]

Specifications table

**Table:** 

Subject	Medicine and Dentistry
Specific subject area	Cardiology and Cardiovascular Medicine
Type of data	Table Figure
How data were acquired	Data were retrospectively and prospectively collected. FACS data were acquired using a FACS Calibur (BD). Calculations were conducted with IBM SPSS version 18. Graphics were designed by using IBM SPSS version 18 or Sigma Plot 10.0.
Data format	Raw Analyzed
Parameters for data collection	Patients with symptomatic severe aortic stenosis, undergoing transcutaneous valve replacement, were consecutively enrolled at our departement of cardiology and internal medicine at Technische Universität Dresden, Heart Center Dresden, University Hospital, Germany.
Description of data collection	Peripheral venous blood samples were collected from all participants through non-traumatic puncture and minimal stasis into sodium-citrate containing tubes and analysed by flow cytometry within 60 minutes after collection. In total, flow-cytometric quantification analyses measurements by cytometric bead arrays (CBA) or routine laboratory tests were performed in 120 patients before and the days after TAVI.
Data source location	Institution: Technische Universität Dresden, Heart Center Dresden, University Hospital City/Town/Region: Dresden Country: Germany
Data accessibility	With the article
Related research article	C. Pfluecke, S. Wydra, K. Berndt, D. Tarnowski, M. Cybularz, S. Jellinghaus, J. Mierke, G. Ende, DM. Poitz, P. Barthel, FM. Heidrich, KM. Sveric, S. Quick, U. Speiser, A. Linke, K. Ibrahim, Mon2-Monocytes and Increased CD-11b Expression Before Transcatheter Aortic Valve Implantation are Associated with Earlier Death, Int J Cardiol. In Press.

## Value of the data

• CD11b-expression on monocytes as sign of enhanced cellular activity on day one after TAVI is associated with early death after TAVI.
• Determination of parameters of cellular activity or inflammatory cells, like monocyte subpopulations, help to better understand the mechanism of the SIRS after TAVI
• The presented data of an association of monocyte activity to mortality and to parameters of platelet activation and further inflammatory marker could be used for further studies to acquire insight into possible underlying causal pathophysiological mechanism after TAVI.
• Parameters, like CD11b expression on monocytes, or IL-8 may disclose a potential use as biomarkers or function as possible therapeutic targets in the future.

## 1 Data Description

We present data of 120 symptomatic patients with severe aortic stenosis, who underwent TAVI with a transfemoral approach. We focused on parameters of inflammatory reactions after TAVI, which are suspected to have negative effects for the outcome of the patients.[2] We also present data on monocyte subpopulations, which could be associated with cardiovascular events [3], MPAs, as established marker for platelet activation [4] and CD11b expression as an marker of monocyte activation, which could be already shown in patients with atrial fibrillation [5] and thrombogenicity [6].

Table 1 shows procedural outcomes and mortality at 30 days in dependence of three-month mortality. The data show, that there was only a small number of direct procedure-related complications after TAVI. Within 30 days after TAVI, three strokes occurred in the group with worse outcome compared to one stroke in the survivor group. The distribution of diagnosed post-procedural infections was comparable in both groups. The proportion of patients with increased body temperature in the first two days after TAVI was equally distributed. Among patients, who died within the first three months after TAVI, only two died within the first 30 days after TAVI.

**Table 1:** Procedural Outcomes and Mortality at 30 Days in dependence of three-month mortality

Parameter	survivors after 3 months(n= 105)	non-survivors after 3 months(n=15)	p Value
Balloon expendable valve (%)	10 (9.5)	1 (7)	0.723
Self-expendable valve	95 (90.5)	14 (93)	0.723
Device Success (transfemoral)	105 (100)	14 (93)	0.334
Residual aortic regurgitation ≥2	6 (6)	4 (27)	0.105
Residual aortic peak velocity, m/s	0.198 ± 0.048	0.194 ± 0.053	0.751
Residual aortic mean gradient, mmHg	8.7 ± 4.6	9.9 ± 5.8	0.440
≥ 38°C on day 1 after TAVI n (%)	39 (37)	3 (20)	0.204
≥ 38°C on day 2 after TAVI n (%)	23 (22)	3 (20)	0.542
**VARC-2**			
Myocardial infarction	0 (0)	0(0)	1
Stroke	1 (1)	3 (20)	0.006#
Renal Failure	3 (3)	1 (3)	0.507
Bleeding	25 (23.8)	5 (33)	0.426
Access site complication	9 (8.6)	1 (7)	0.803
New pacemaker / ICD	32 (30.5)	6 (40)	0.507
Mortality 30d	0	2(13)	0.015#
post-procedure infections n (%)	28 (27)	4 (27)	1.0

TAVI, transcatheter aortic valve implantation, VARC-2, Valve Academic Research Consortium-2, ICD, implantable cardioverter-defibrillator. # the observed events are less than 5.

In Table 2, systemic parameters of inflammation in dependence of three-month mortality in the first days after TAVI were presented. Those who did not survive showed a significant increase in CRP and IL-8, especially the day before TAVI. In contrast, the CRP values in the days after TAVI, in which period SIRS typically occurs [2], showed no association with early mortality. Only IL-8 on day 1 after TAVI and procalcitonin 3 days after TAVI showed a slight association with early mortality. Neither the number of infections after TAVI nor the procalcitonin values before TAVI and in the first two days after TAVI differed between survivors and died patients. Therefore, we do not assume that SIRS or sepsis directly after TAVI in our patient cohort had a decisive influence on three-month mortality.

**Table 2:** Systemic parameters of inflammation in dependence of three-month mortality

Parameter	survivors after 3 months (n= 105)	non-survivors after 3 months (n=15)	P Value
leukocytes d0, Gpt/l	6.9 (5.9-8.3)	7.3 (6.5-8.4)	0.387
*leukocytes d1, Gpt/l*	8.9 (7.4-11.1)	8.7 (7.4-9.4)	0.396
*leukocytes d2, Gpt/l*	7.8 (6.6-9.9)	8.4 (6.6-10.5)	0.882
*leukocytes d3, Gpt/l*	7.8 (6.4-9.8)	8.5 (6.5-10.8)	0.378
*leukocytes d4, Gpt/l*	7.2 (6.1-8.8)	7.1 (6.0-9.5)	0.979
leukocytes d5, Gpt/l	6.8 (5.6-8.5)	8.1 (6.1-9.8)	0.058
*CRP d0, mg/l*	3.9 (1.6-6.2)	6.8 (5.0-12.5)	0.029*
*CRP d1, mg/l*	15.6 (9.5-26.9)	14.7 (10.8-49.7)	0.670
*CRP d2, mg/l*	59.0 (35.4-84.0)	58.0 (41.5-108.5)	0.569
*CRP d3, mg/l*	69.0 (48.2-99.4)	78.4 (47.9-112.0)	0.570
*CRP d4, mg/l*	67.3 (38.0-109.0)	60.9 (51.6-146.0)	0.459
*CRP d5, mg/l*	53.1 (33.1-100.0)	54.2 (35.7-111.5)0.	0.640
IL-6 d0, ng/l	3.2 (1.8 - 5.2)	4.6 (3.9 - 9.1)	0.083
IL-6 d1, ng/l	62.0 (40.7-94.1)	45.5 (34.0-143.0)	0.793
IL-6 d3, ng/l	40.4 (26.0-65.6)	62.3 (40.3-132.0)	0.100
IL-6 d7, ng/l	1.3 (0-10.8)	8.1 (0-17.5)	0.305
IL-8 d0, ng/l	5.9 (3.8-8.5)	9.8 (7.0-12.3)	0.005**
IL-8 d1, ng/l	13.6 (9.5-19.2)	18.7 (14.2-25.8)	0.026*
IL-8 d7, ng/l	13.0 (7.5-18.2)	9.4 (8.8-12.0)	0.393
PCT d0, µg/l	0.1 (0.06 - 0.10)	0.1 (0.08-0.10)	0.231
PCT d1, µg/l	0.1 (0.10 - 0.16)	0.13 (0.10 - 0.31)	0.160
PCT d2, µg/l	0.11 (0.07 - 0.19)	0.20 (0.15 - 0.20)	0.332
PCT d3, µg/l	0.1 (0.09 - 0.15)	0.18 (0.10 - 0.56)	0.036*
PCT d5, µg/l	0.1 (0.09 - 0.18)	0.12 (0.10 - 0.25)	0.167
neutrophils d0, Gpt/l	4.2 (3.4 – 5.5)	4.7 (4.1 – 5.5)	0.312
neutrophils d1, Gpt/l	6.7 (5.4 – 8.3)	7.1 (5.6 – 7.7)	0.374
neutrophils d5, Gpt/l	4.4 (3.5 – 6.1	5.8 (4.6 – 8.3)	0.832
lymphocytes d0, Gpt/l	1.6 (1.2 – 1.9)	1.6 (1.3 – 2.2)	0.386
lymphocytes d1, Gpt/l	0.9 (0.7 – 1.2)	0.8 (0.5 – 1.1)	0.029*
lymphocytes d5, Gpt/l	1.1 (0.9– 1.4)	0.8 (0.7 – 1.4)	0.283

CRP, C-reactive protein; IL, Interleukin, PCT, Procalcitonin, * p<0.05, ** p<0.01.

In Table 3, parameters of flow cytometry on day 1 after TAVI in dependence of three-month mortality were shown. The MFI of CD16 on monocytes is significantly associated with mortality, whereas the absolute count of MON2 (CD14++/CD16+) is elevated by trend in the group of the non-survivors. In contrast, MON1-monocytes (CD14++/CD16-) showed a significant association with survival, which suggests that the kind of inflammation and the involved monocyte subpopulations rather than the extent of inflammation itself determines the survival. Furthermore, the expression of the active epitope CD11b on monocytes showed a significant association with early dead after TAVI.

**Table 3:** Parameters of flow cytometry on day 1 after TAVI in dependence of three-month mortality

Parameter	survivors after 3 months (n=93)	non-survivors after 3 months (n=12)	P Value
Mon1 cells/µl	591 (441-826)	422 (346-529)	0.007**
Mon2 cells/µl	62 (41-100)	91 (66-106)	0.101
Mon3 cells/µl	22 (12-40)	21 (14-43)	0.513
MFI CD16 on monocytes	22 (17-32)	38 (23-46)	0.007**
CD11b+ monocytes, cells/µl	35 (23-58)	54 (18-123)	0.514
MFI CD11b on monocytes	23 (17-39)	42 (30-60)	0.007**
MPA, cells/µl	237 (160-326)	206 (153-329)	0.481

Mon1 (CD14^++^/CD16^−^), Mon2 (CD14^++^/CD16^+^), Mon3 (CD14^+^/CD16^++^), MFI, mean fluorescence intensity, MPA, Monocyte Platelet Aggregates, * p<0.05, ** p<0.01.

In Table 4, we present data of flow cytometry on day 7 after TAVI in dependence of three-month mortality. Here, we see an association of Mon2 and of CD11b expression in monocytes with mortality by trend, without reaching level of significance.

**Table 4:** Parameters of flow cytometry on day 7 after TAVI in dependence of three-month mortality

Parameter	survivors after 3 months (n=56)	non-survivors after 3 months (n=10)	P Value
Mon1 cells/µl	408 (312-548)	510 (461-560)	0.058
Mon2 cells/µl	37 (26-56)	57 (33-86)	0.111
Mon3 cells/µl	16 (10-32)	31 (18-45)	0.058
MFI CD16 on monocytes	18 (14-24)	24 (19-32)	0.088
CD11b+ monocytes, cells/µl	31 (16-56)	58 (22-161)	0,084
MFI CD11b on monocytes	24 (15-48)	38 (23-51)	0.112
	200 (140-306)	170 (136-260)	0.604

Mon1 (CD14^++^/CD16^−^), Mon2 (CD14^++^/CD16^+^), Mon3 (CD14^+^/CD16^++^), MFI, mean fluorescence intensity, MPA, Monocyte Platelet Aggregates.

**Fig. 1:**
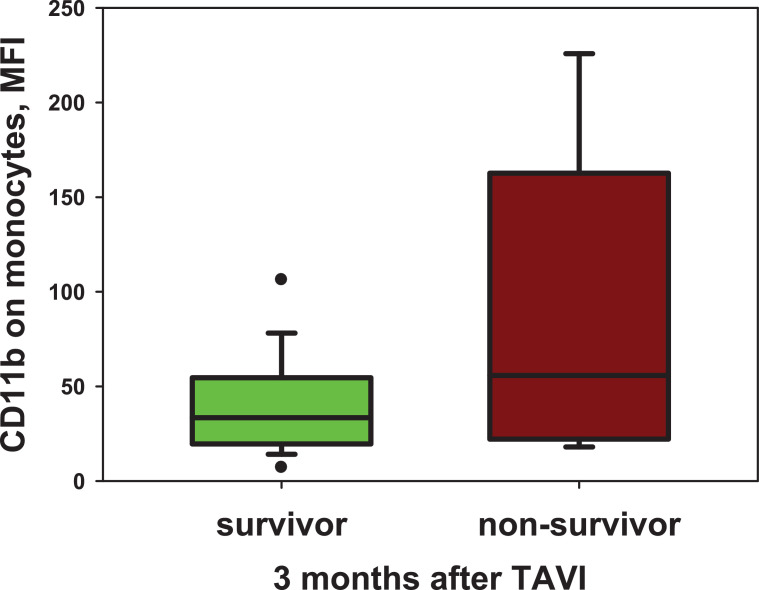
The CD11b-expression on monocytes in dependence of three-month survival after TAVI The mean fluorescence intensity (MFI) of CD11b on monocytes before TAVI in comparison of survivor (n=105) with non-survivor (n=15) three months after TAVI. Data are presented as box and whisker plots. The ends of the whiskers represent the 5th and the 95th percentile. p=0.024

Fig. 1 illustrates the significant association of CD11b expression on monocytes before TAVI with early mortality afterwards.

**Fig. 2:**
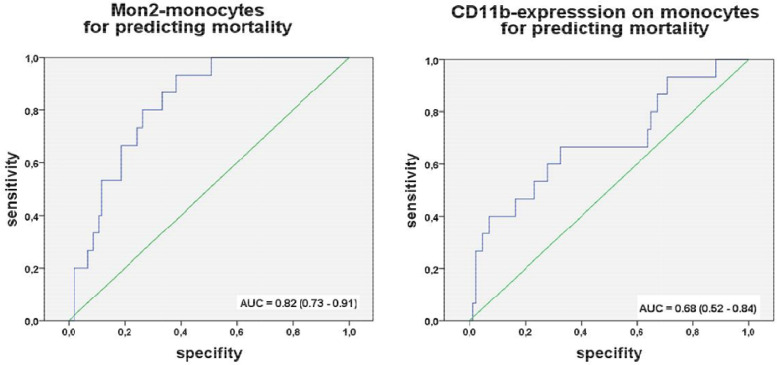
Receiver operating characteristic (ROC) curve analyses of the absolute count of Mon2 (left) and the CD11b-expression on monocytes (right) on day before TAVI for predicting three-month mortality after TAVI AUC indicates area under the curve. The number in parentheses indicates 95% confidence of intervals.

In Fig. 2 we demonstrate receiver operating characteristic (ROC) curve analyses of the absolute count of Mon2 (left) and the CD11b-expression on monocytes (right) on day before TAVI for predicting three-month mortality after TAVI. For Mon2 the ROC-analyses revealed an area under the curve (AUC) of 0.82 (95% CI 0.73-0.91) for Mon2 monocytes before TAVI in predicting mortality afterwards. Mon2 monocytes above >50 cells/µl predicted mortality with a sensitivity of 73% and a specificity of 80%. For the CD11b expression on monocytes, the ROC-analyses revealed an odds ratio of 5.0 (95% CI 1.1 – 22.0, p=0.033) if the average MFI of CD11b-expression on monocytes was above 55.

**Fig. 3:**
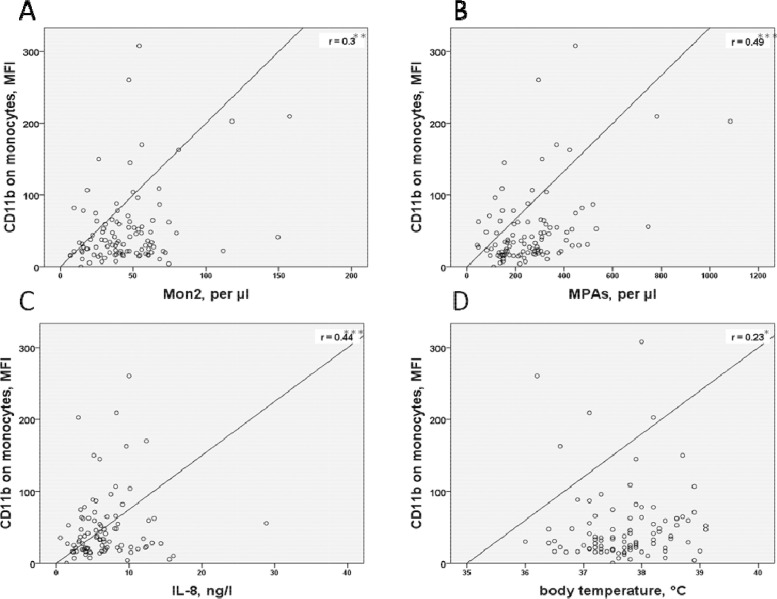
Correlation analysis of CD11b-expression on monocytes the day before TAVI with: A, MON2 content on day before TAVI; B, the content of MPAs on day before TAVI; C, IL-8 on day before TAVI; D, the body temperature on day one after TAVI. Mon2, (CD14++/CD16+); MFI, mean fluorescence intensity, MPA, Monocyte platelet Aggregates; IL-8, Interleukin 8. * p<0.05, ** p<0.01, *** p<0.001.

Fig. 3 illustrates the significant correlations, we found between monocyte activation (CD11b expression) and parameters of platelet activation (MPAs), monocyte subpopulation (Mon2 content), systemic inflammatory cytokine (IL-8) on day before TAVI and to a clinical SIRS-parameter (body temperature) on day one after TAVI.

Individual raw data on procedural outcome and on all measured parameters are listed in a supplementary Excel sheet.

## 2 Experimental Design, Materials and Methods

In 120 patients with symptomatic aortic stenosis, flow cytometric analyses were performed one day before TAVI, on day one after TAVI and seven days after TAVI. Therefore, peripheral venous blood samples were collected from all the study participants through non-traumatic puncture and minimal stasis into sodium-citrate containing tubes and analyzed by flow cytometry within 60 minutes of collection. In total, flow-cytometric quantification analyses were performed in 120 patients. A few patient data are missing on day 1 and day 7 after TAVI, as can be seen from the corresponding tables. Shortly after, 50 µl blood samples were labeled within 10 min with CD45-FITC, CD14-APC, CD11b-PE and CD41-PE (all antibodies were purchased from Becton Dickinson [BD], Oxford, United Kingdom). CD11b-PE and CD41-PE were used in different tubes. As control for the determination of CD11b on monocytes, tubes containing CD45, CD14 but not CD11b (fluorescence minus one) were used. Flow cytometric measurements were performed using the BD FACSCalibur flow cytometer. Monocytes were identified by gating strategies based on CD45-expression and side scatter to select monocytes. The degree of co-expression of CD16 and CD14 on monocytes was determined. Subsets were defined as Mon1: CD14++CD16– (“classical”), Mon2: CD14++CD16+ (“intermediate”), and Mon3: CD14+CD16+ (“nonclassical”) monocytes [7]. The extent of monocyte activation was determined by quantification of the active epitope of the cell adhesion molecule CD11b (MAC-1). The expression of CD11b was measured as mean fluorescence intensity (MFI). The values of fluorescence minus one (FMO) control were subtracted. The content of MPAs was determined by co-expression of CD41 and CD14 on monocytes. Absolute counts of monocyte subpopulations (in cells per µl) were obtained by calculating the number of monocytes proportional to the number of the count in the standard laboratory blood test. For this dual-platform approach, a separate hematoanalyser was used to obtain total leukocyte counts. Proportions of monocyte subsets were obtained by flow cytometry on the same day. IL-6 and IL-8 were assessed by using the cytometric bead arrays (CBA) [8], according to the manufacturer's instructions. Therefore, blood samples were collected in sodium citrate tubes and plasma prepared by centrifugation at 2000xg for 10 min at 4°. The plasma aliquots were stored at -80°C until assayed. Additionally, C-reactive protein and procalcitonin were measured as part of the routine laboratory tests.

## 3 Ethics Statement

The study was performed in accordance with the Helsinki Declaration and approved by the institutional ethics committee of the Technische Universität Dresden (EK 406122012). All the patients participated voluntarily and gave their written, informed consent.

## Declaration of Competing Interest

The authors declare that they have no competing financial interests or personal relationships which have, or could be perceived to have, influenced the work reported in this article.

## Appendix Supplementary materials

Image, application 1
